# Hereditary Elliptocytosis Identified During the Evaluation of Splenomegaly: A Case Report

**DOI:** 10.7759/cureus.114970

**Published:** 2026-08-22

**Authors:** Bonaventure Oguaju, Marlene Hamilton, Lindsay Bridgland

**Affiliations:** 1 General Internal Medicine, University of Alberta, Edmonton, CAN; 2 Hematology, University of Alberta, Edmonton, CAN

**Keywords:** anemia, hereditary elliptocytosis, jaundice, mcv, splenomegaly

## Abstract

Hereditary elliptocytosis (HE) is a red blood cell (RBC) membrane disorder resulting from a mutation in RBC membrane proteins that predispose the cell to hemolysis. Clinical presentation can range from patients being asymptomatic to hemolysis with splenomegaly and anemia.

In this report, we present the case of a 40-year-old female with a history of intermittent normocytic normochromic anemia and chronic left upper abdominal pain with mild splenomegaly. During workup for splenomegaly, she was found to have elliptocytes on her blood smear, leading to a clinical diagnosis of HE. For individuals diagnosed with asymptomatic HE, the management focus should be on counseling about the condition, including education on the complications associated with HE.

## Introduction

Hereditary elliptocytosis (HE) is an inherited red blood cell (RBC) disorder defined by elongated, oval, or elliptical-shaped RBCs on the peripheral blood smear. Elliptocytosis was first described by Dresbach in 1904, and its hereditary nature was established by Hunter [1].

Subtypes of HE include common HE, hereditary pyropoikilocytosis (HPP), Southeast Asia ovalocytosis (SAO), and spherocytic elliptocytosis (SE). The subtypes have varied RBC morphology and degrees of hemolysis [1]. The inheritance pattern for most forms of HE is autosomal dominant, except HPP, which is inherited in an autosomal recessive manner [2,3].

The global prevalence of HE is 1 in 2000 to 1 in 4000 individuals, but the prevalence is higher in certain West African regions, including the Republic of Benin and Togo, approximately 1 in 100 individuals [4]. The various subtypes of HE are associated more commonly with different ethnic backgrounds. HPP, the more severe variant of HE, affects less than 10% of individuals [1]. The prevalence rate of SAO ranges between 5% and 25% and is commonly seen in malaria-endemic areas such as Malaysia, Papua New Guinea, Indonesia, the Philippines, and Southern Thailand [4]. SE typically affects individuals of European descent [4,5].

Splenomegaly is typical in patients with HE. The spleen plays an essential role in this disorder by entrapping and eliminating the abnormal elliptocytes, resulting in hemolytic anemia [1]. Most patients with HE are asymptomatic and do not need any treatment. Symptomatic patients may require interventions, including blood transfusion and splenectomy [6,7].

In this report, we present the case of a female patient from Canada diagnosed with HE while being evaluated for splenomegaly.

## Case presentation

A 40-year-old female from western Canada with a medical history significant for intermittent mild anemia, splenomegaly, and previous atrio-ventricular re-entrant tachycardia (status post atrio-ventricular node ablation) was assessed in the General Internal Medicine (GIM) outpatient clinic for evaluation of splenomegaly. She initially presented to the Emergency Department with a one-year history of left upper abdominal and left shoulder pain. CT abdomen done in the emergency room showed progression of splenomegaly compared to an ultrasound performed one year earlier. She was thus referred for further assessment. She reported early satiety, fatigue, and a 25-pound unintentional weight loss over seven months. Physical examination was normal, including a negative Castell’s sign.

**Figure 1:**
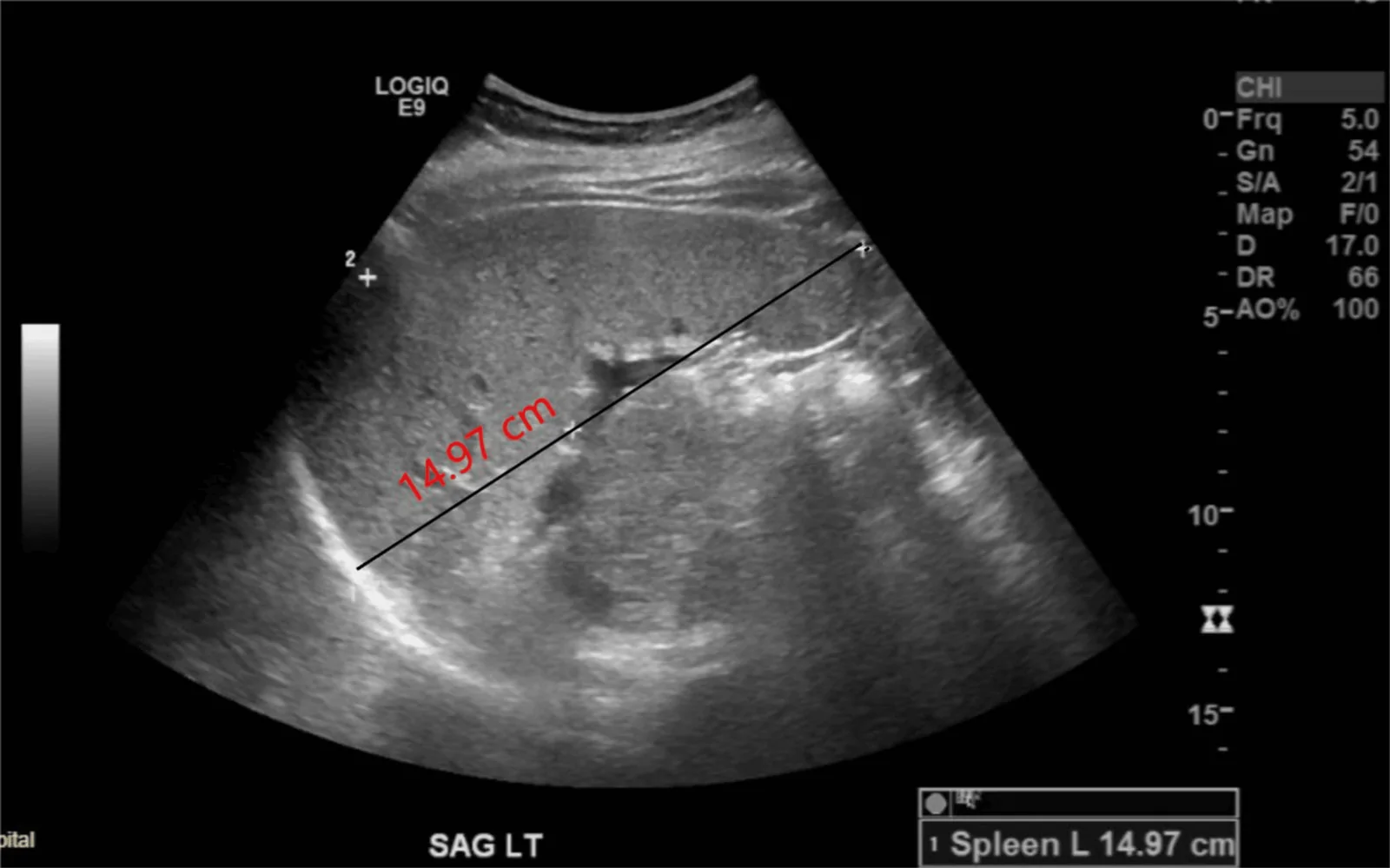
Splenomegaly demonstrated on ultrasound The black line marks the long axis of the spleen, measuring 14.97 cm.

Esophagogastroduodenoscopy demonstrated mild gastritis. Repeat abdominal ultrasound was performed, confirming persistent splenomegaly with splenic size of 15 cm measured craniocaudally. There were no gallstones or features of portal hypertension (Figure 1).

Complete blood count (CBC) revealed normal hemoglobin 130 g/L, mean corpuscular volume (MCV) 88 fL, white blood cell count 5.1, and platelets 203 (Table 1). The hemolytic markers, including bilirubin 12 umol/L, lactate dehydrogenase (LDH) 197 u/L, reticulocyte count 1.5%, and haptoglobin 0.49 g/L, were normal (Table 2). Serum vitamin B12 was normal at 374 (normal range ≥ 160 pmol/L), and serum ferritin level was at the lower end of normal at 20 (normal range 20-300 ug/L). With regard to the splenomegaly, autoimmune, amyloidosis, and infectious work-ups were negative. Peripheral blood flow cytometry did not detect any immunophenotypic abnormality within the lymphoid population.

**Table 1:** Complete blood count

Test	Result	Reference range (units)
White blood cell	5.1	4.0-11.0 (10^9^/L)
Red blood cell	4.50	3.80-5.20 (10^12^/L)
Hemoglobin	130	120-160 (g/L)
Hematocrit	0.40	0.36-0.48 (L/L)
Mean corpuscular volume	88	80-100 (fL)
Mean corpuscular hemoglobin concentration	327	310-360 (g/L)
Red blood cell distribution width	13.8	<16.0 (%)
Platelets	203	140-400 (10^9^/L)

**Table 2:** Hemolytic markers

Test	Result	Reference range (units)
Bilirubin	12	<20 (umol/L)
Lactate dehydrogenase	197	120-250 (U/L)
Reticulocyte count	1.5	0.4-2.0 (%)
Haptoglobin	0.49	0.3-2.00 (g/L)

**Figure 2:**
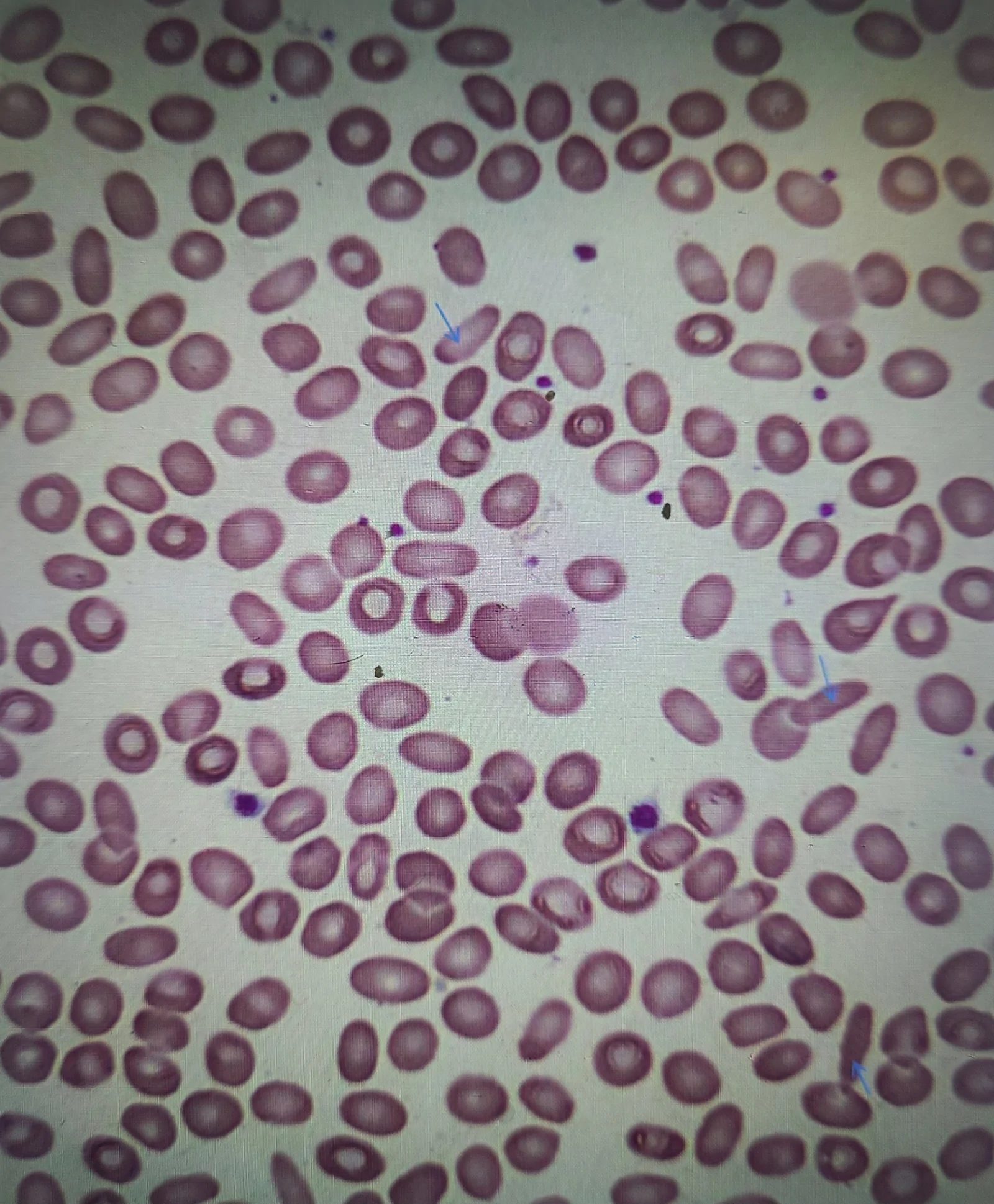
Elliptocytes on peripheral blood smear (thin blue arrow) Wright-Giemsa-stained; 100× magnification.

However, peripheral blood smear showed normocytic anemia without significantly increased polychromasia, with many elliptocytes. Platelets and leukocytes were morphologically unremarkable (Figure 2). Other causes of elliptocytosis were considered, including myelodysplastic syndrome, but she did not have pancytopenia or macrocytosis.

In view of the persistent mild splenomegaly, history of intermittent normocytic anemia, no family history of HE, and findings of many elliptocytes in the peripheral blood smear with no biochemical evidence of hemolysis, she was referred to the hematology clinic. Following her assessment, a clinical diagnosis of HE was made, with a possibility of intermittent low-grade hemolysis.

After the diagnosis, the patient was counseled about the condition and was recommended to get an abdominal ultrasound every three to four years for cholelithiasis screening.

## Discussion

HE results in the formation of elliptocytes with reduced lifespan of the RBCs [8]. HE results from genetic mutations affecting the RBC cytoskeleton proteins, including α-spectrin (SPTA1), β-spectrin (SPTB), protein 4.1 (EPB41), band 3, and glycophorin C [4,9]. The SPTA1 mutation is the most common mutation in HE, seen in 65% of cases, followed by SPTB mutations (30%) and EPB41 mutations (5%) [1]. These genetic alterations affect RBC membrane function and shape, resulting in fixed morphology in the form of elliptocytes. The abnormal elliptocytes can be captured and eliminated by the spleen, leading to a reduction in RBC lifespan and hemolytic anemia.

HE can be classified based on their manifestations into three classes: asymptomatic, hemolytic compensatory, and hemolytic anemia. Classification is based on the patient’s hemoglobin, reticulocyte count, presence or absence of jaundice, and degree of splenomegaly [10]. Asymptomatic and hemolytic compensatory types do not usually cause symptoms or signs of anemia or jaundice and may not have any abnormal RBCs on a peripheral blood smear [11]. The hemolytic anemia types usually have low hemoglobin, increased reticulocyte count, and an increase in abnormal RBCs in the peripheral blood [11]. Most patients are asymptomatic and are found incidentally during workup for anemia. Symptomatic patients may present with signs of hemolytic anemia, including fatigue and jaundice. They may also present like our index patient, with clinical signs related to splenomegaly, including left upper quadrant pain, early satiety, and referred left shoulder pain.

Workup for suspected HE includes CBC, which may show normocytic normochromic anemia. MCV is typically low with some subtypes of HE, specifically hemolytic HE and HPP. These variants cause RBC fragmentation, leading to severe microcytosis [12]. Hemolytic markers, including elevated LDH, elevated indirect bilirubin, elevated reticulocyte counts, and reduced haptoglobin level, may be abnormal.

A peripheral blood smear is essential for the diagnosis of HE. The blood smear typically shows elliptocytes in 15% to 100% of RBCs, along with other variations, including spherocytes, stomatocytes, poikilocytes, ovalocytes, and macro-ovalocytes [12,13]. The preponderance of elliptocytes does not correlate with the degree of hemolysis [9]. Sodium dodecyl sulphate-polyacrylamide gel (SDS-PAGE) can be used for detailed analysis to measure the RBC membrane proteins, like spectrin and protein 4.1 [1]. Abdominal ultrasound can be used for assessment of splenomegaly when it is suspected. It is important to educate patients and their families about the inheritance patterns of the condition. Genetic testing is essential for confirmation of diagnosis, management, and genetic counseling, especially in individuals with atypical presentation [13].

Asymptomatic individuals without hemolysis do not need any intervention or regular follow-up. Patients with hemolysis or anemia may require blood transfusion, and splenectomy may be required for severe, life-threatening anemia or individuals requiring frequent blood transfusions [1].

Complications of HE include megaloblastic anemia, cholelithiasis (leading to cholangitis, cholecystitis, or pancreatitis), splenomegaly, renal tubular acidosis (associated with SAO), and skeletal abnormalities from bone marrow expansion (commonly seen in HPP) [1].

## Conclusions

HE is a rare genetic disorder in Canada. There are no available Canadian prevalence statistics, but the global prevalence may be underestimated given that most patients are asymptomatic. Most individuals diagnosed with HE, including our patient, have no symptoms of hemolysis and require supportive care with routine monitoring of serum vitamin B12 and folate levels with replacement as needed. These patients also require regular screening for cholelithiasis with an abdominal ultrasound. For patients with splenomegaly, the focus of management is on treating hemolytic anemia with supportive measures and consideration of splenectomy in individuals who require frequent transfusions.
